# When a tree falls: Controls on wood decay predict standing dead tree fall and new risks in changing forests

**DOI:** 10.1371/journal.pone.0196712

**Published:** 2018-05-09

**Authors:** Brad Oberle, Kiona Ogle, Amy E. Zanne, Christopher W. Woodall

**Affiliations:** 1 Department of Biological Sciences, George Washington University, Washington, DC, United States of America; 2 Center for Conservation and Sustainable Development, Missouri Botanical Garden, St. Louis, Missouri, United States of America; 3 Division of Natural Sciences, New College of Florida, Sarasota, Florida, United States of America; 4 School of Informatics, Computing, and Cyber Systems, Northern Arizona University, Flagstaff, Arizona, United States of America; 5 Northern Forest Science and Applications, Northern Research Station, Durham, New Hampshire, United States of America; Pacific Northwest National Laboratory, UNITED STATES

## Abstract

When standing dead trees (snags) fall, they have major impacts on forest ecosystems. Snag fall can redistribute wildlife habitat and impact public safety, while governing important carbon (C) cycle consequences of tree mortality because ground contact accelerates C emissions during deadwood decay. Managing the consequences of altered snag dynamics in changing forests requires predicting when snags fall as wood decay erodes mechanical resistance to breaking forces. Previous studies have pointed to common predictors, such as stem size, degree of decay and species identity, but few have assessed the relative strength of underlying mechanisms driving snag fall across biomes. Here, we analyze nearly 100,000 repeated snag observations from boreal to subtropical forests across the eastern United States to show that wood decay controls snag fall in ways that could generate previously unrecognized forest-climate feedback. Warmer locations where wood decays quickly had much faster rates of snag fall. The effect of temperature on snag fall was so strong that in a simple forest C model, anticipated warming by mid-century reduced snag C by 22%. Furthermore, species-level differences in wood decay resistance (durability) accurately predicted the timing of snag fall. Differences in half-life for standing dead trees were similar to expected differences in the service lifetimes of wooden structures built from their timber. Strong effects of temperature and wood durability imply future forests where dying trees fall and decay faster than at present, reducing terrestrial C storage and snag-dependent wildlife habitat. These results can improve the representation of forest C cycling and assist forest managers by helping predict when a dead tree may fall.

## Introduction

**Fig 1:**
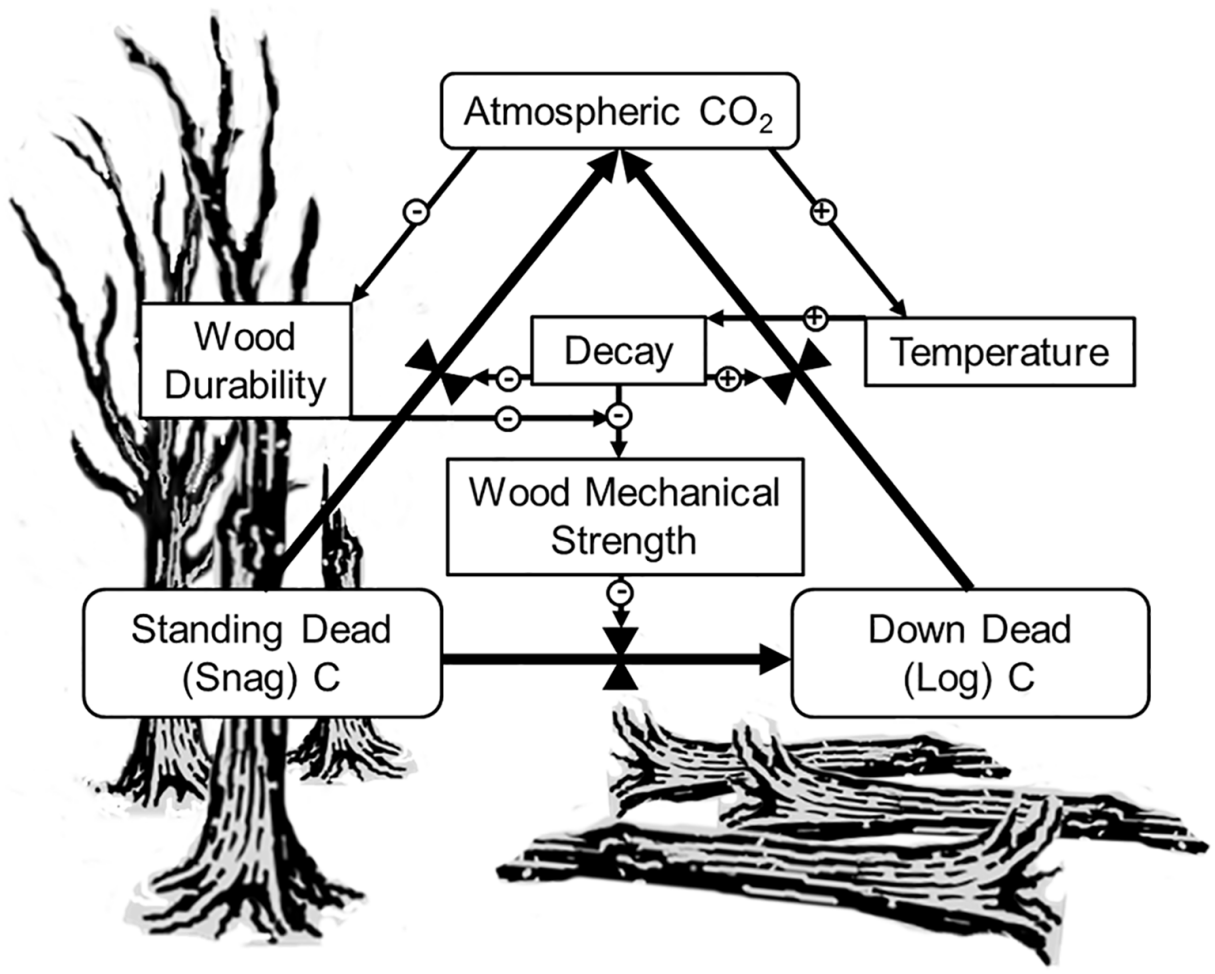
Schematic representation of the deadwood C cycle and variables that affect snag fall rates. C pools are surrounded by rounded boxes and C fluxes by bold arrows. Controlling or predictor variables are in square boxes and their hypothesized influence on fluxes represented by thin solid arrows pointing to control symbols associated with fluxes. The hypothesized direction of the effect is indicated by symbol in the open circle.

Standing dead trees, also called snags, play pivotal roles in the structure and function of changing forests (Fig 1). Snags are a keystone structure for many endangered species, providing such important wildlife habitat that forest management guidelines often set explicit targets for minimum snag density [1]. Snags also represent a major aboveground carbon (C) pool, accounting for over 1 Pg C in the United States alone [2]. Regional variation in snag C closely tracks climate change-related forest disturbances [3]. Drought, fire, and beetle outbreaks all transform productive aboveground biomass into standing deadwood. Whether forest dieback tips forests from net C sinks to sources reflects, among other factors, how snag formation and fall influences deadwood decay [4]. While deadwood is suspended off the ground, desiccation and nutrient limitation slow both decomposer activity and the rate of decomposition-derived greenhouse gas emissions to the atmosphere [5,6]. In this state, snags can delay the C efflux following disturbance for many years. For example, C emissions from an Oregon forest decreased following a bark beetle outbreak because dead trees no longer stimulated soil respiration and decomposed very slowly [7]. However, once snags fall to the forest floor and become logs, increased soil contact accelerates saprotrophic respiration by providing a more stable, moist, and accessible environment for decomposers [8]. Differences in decay rates between standing and down deadwood can be dramatic. In a recent long-term experiment [9], ground contact accelerated wood decay by an order of magnitude, which is comparable to the difference in decay rates between leaf litter and wood of the same species [10]. Consequently, snag fall is a primary control on net forest C balance in the years to decades following disturbance [4,11,12].

In addition to their pivotal role in the forest C cycle, standing dead trees threaten public safety and property. Hazard trees, including snags, are a major source of litigation in the United States where falling trees kill more than 200 people each year [13,14]. Many fatalities occur among tree-care professionals who are tasked with protecting both people and property. As global change stresses aging trees in publically accessible forests, management agencies are allocating an increasing portion of limited budgets to hazard tree identification and removal [15]. Understanding drivers of snag dynamics in unmanaged forests may translate into improved hazard tree risk assessments for managed forests, helping decision makers allocate resources to removal in the short term and plan for safer forests in the long term.

Evaluating the impacts of snag dynamics in changing forests requires improving predictions for when snags fall based on a mechanistic understanding of how they fall [11,16,17]. As with any structural failure, snags fall when applied mechanical stress exceeds toughness [18]. Snag toughness depends in part on wood density, and wood density generally declines as wood decays [19]. Therefore, snag fall rates should depend on two kinds of factors: those that expose trees to breaking forces and those that accelerate wood decay. Factors that expose trees to breaking forces represent an interaction between the tree architecture and mechanical loadings from wind, ice, and water [18]. Factors that influence wood decay rates vary with intrinsic features of deadwood, such as initial density, chemistry, and decomposer communities, as well as extrinsic features such as temperature and soil moisture [20]. Whether exposure to stress or wood decay more strongly influences snag fall rates has important implications for predicting how snags’ roles may shift in changing forests. For instance, if snag fall rates depend primarily on factors than limit wood decay, several positive feedbacks could intensify forest C cycling (Fig 1). Quantifying this risk requires identifying how different external and intrinsic factors govern snag fall.

Many factors that influence snag fall rates vary with features of the environment where snags form. Regional climate, topography, and stand-level factors may all play roles. Across broad geographic gradients, climatic variation can influence both exposure to mechanical stress and wood decay. Higher wind loads promote tree fall [18,21] and increasing temperature, among other factors, weakens wood by accelerating decay [22,23]. Regional variation in climate may explain why calm, cool forests at high latitudes in North America store relatively more C in standing deadwood [24]. At the landscape scale, topography also plays a role [25]; snag fall rates may increase where steep slopes or flooding destabilize soils and where high soil moisture accelerates decay [20]. For example, over a 15 year interval, ponderosa pine snag fall rates increased with slope and topographic exposure in northern Arizona [26]. Within landscapes, dense stands with large trees may shield individual snags from wind [11,18]. However, in certain settings, dense stands can promote snag fall via a domino effect [27], or by increasing the prevalence of wood decay fungi [28].

Besides factors that vary with a snag’s surroundings, snags vary individually in many key ways that may influence how quickly they fall. Existing studies consistently recover effects for tree species identity, stem size, and existing decay [28–32]. The effect of tree species identity may reflect variation in wood traits [19,33]. Some species grow denser and therefore tougher wood [34]. Others imbue non-functional xylem with decay-limiting chemicals, creating naturally durable heartwood that maintains structural integrity well after tree death [35,36]. The same properties can influence variation in snag fall rates within species [30,32]. Wood toughness and stem diameter determine the maximum stress a tree can withstand before breaking [18]. Wider stems also may have proportionally more heartwood which resists decay more effectively than sapwood [35]. Both factors may explain why large snags tend to fall at slower rates [11,31]. Besides wood traits and geometry, many other factors potentially influence wood decomposition. Recent studies have highlighted how the identity, assembly, and activity of decomposer communities can influence decay rates and, depending on context, interact strongly with both wood traits and microclimate [37]. No matter how large, dense, or durable a snag may be, the activity of deadwood feeding organisms erodes mechanical resistance to the point where snags can no longer support their own weight and they fall [18].

Despite growing interest in how snag fall affects changing forests, the relative strength of key drivers across scales is unclear because existing analyses have focused on limited areas. According to a recent review, most tree fall studies have assessed dynamics in particular regions [21]. Studies that estimated snag fall rates specifically have tended to be even more narrowly focused. Most evaluated the persistence of a few thousand snags generated by a major disturbance event in a single forest type in western or northern North America [28–31,38,39]. While these targeted studies have generated many important insights, none has analyzed the large number of snags and potential drivers across the broad geographic gradients necessary to more generally assess snag fall dynamics. The most extensive recent analysis [11] compared dynamics in 10,000 permanent plots across Canada and found considerable variation in snag fall rates by ecozone and dominant species type. However, this analysis did not evaluate the relative roles of different drivers across scales.

To identify broad-scale drivers of variation in snag fall rates, we modelled snag persistence in a forest inventory of almost 100,000 revisited snags, representing over 200 tree species growing from boreal to subtropical forests in over 30,000 plots across the eastern United States. We focused on comparing the strength of factors that control wood decay relative to those that expose snags to breaking forces. Specifically, we predicted that warmer temperatures, mesic hydrology, lower wood durability, and narrower stem diameters would accelerate snag fall by increasing wood decay, while high wood density, calm winds, gradual slopes, and dense surrounding stands would decrease snag fall by limiting the impact of breaking forces. We tested these predictions using a hierarchical model that linked the forest inventory data to independent datasets of species’ wood traits and climate. We used the data-informed model to project changes in snag fall rates as a function of changes in key drivers that are anticipated by mid-century. Finally, we incorporated snag fall projections into a simple forest C model to quantify the effect of temperature driven change on structure and function of an intensively studied forest.

## Results

Among 99,213 standing dead trees observed in 31,411 locations spanning the eastern United States, 48.2% had fallen before resurvey, which occurred 5.01 ± 0.002 (s.e.) years later. The model correctly predicted 66.5% of all observations, improving the prediction odds ratio by a factor of four (95% CI = [3.874, 4.086], p < 0.001). The annualized baseline probability of a snag remaining standing in the eastern United States from 2000–2010, which corresponds to the inverse logit of the intercept term, *β*_*o*_, in Eq 2, was 0.881 (95% CI = [0.871, 0.891]). For 14,251 snags at the earliest stage of decay, only 41.6% had fallen 5.00 ± 0.006 (s.e.) years later, and the model was even more accurate, correctly predicting 72.1% of observations with an estimated prediction odds ratio of 6.46 (95% CI = [5.97, 6.98], p < 0.001). The average recently dead tree in eastern North America had a 50/50 chance of remaining standing (“half-life”) for 7.23 years (95% CI = [6.21, 8.42]yr). These estimates reflect the persistence times solely for standing dead trees and not for living trees that might fall during wind throw or other catastrophic events.

**Fig 2:**
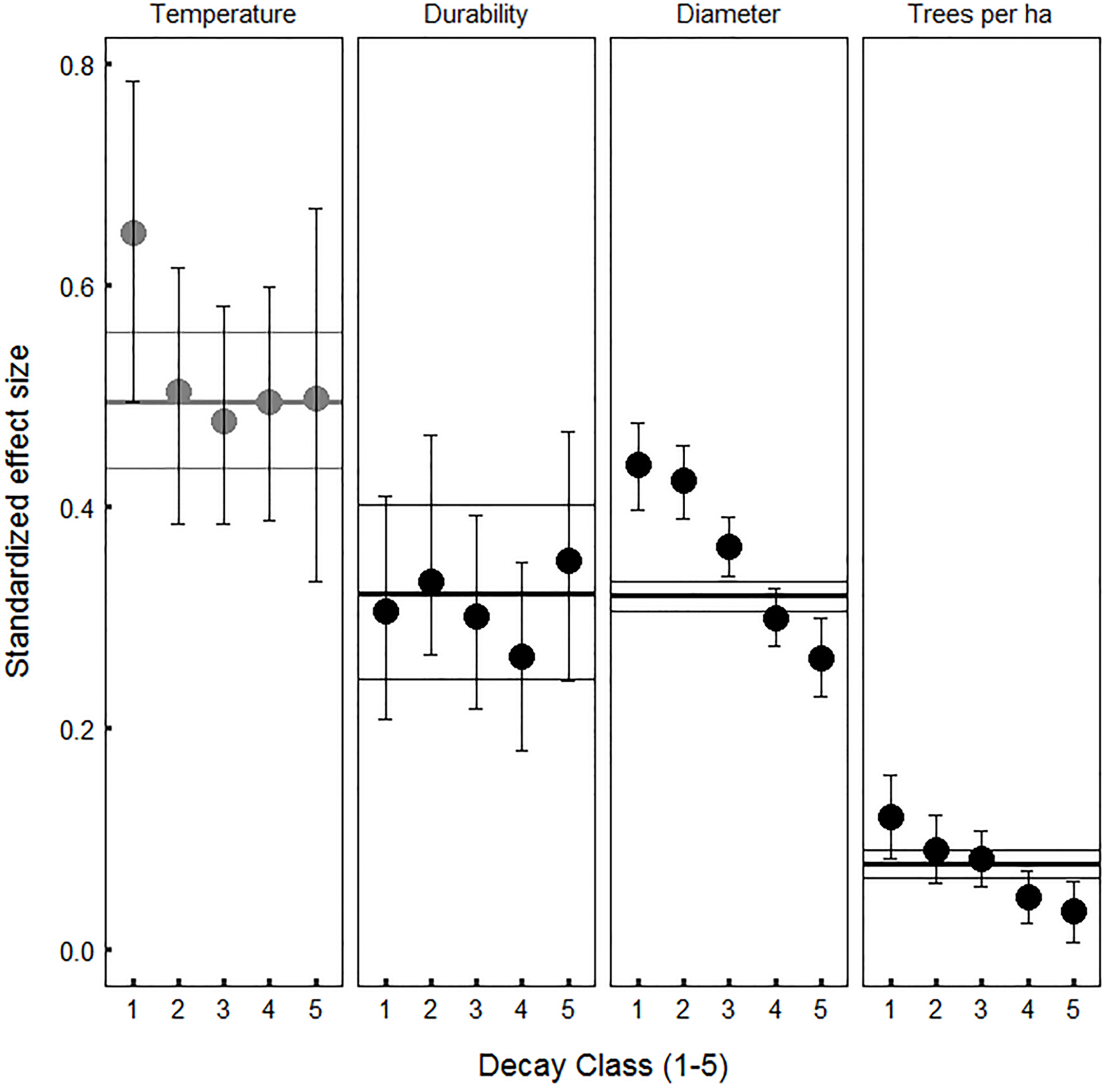
Factors that influence wood decay—temperature, wood durability and initial diameter—strongly affect snag persistence compared to stand density, which primarily influences snag exposure to breaking forces. Predictors include mean annual temperature, species wood durability, initial diameter at 1.37 m height, and number of trees per hectare. Horizontal lines show average effect sizes for all snags and filled circles show effect sizes for subsets representing different progressive decay classes. Outer horizontal lines represent 95% credible intervals (CIs) for all snags. Thin vertical lines (whiskers) with filled circles represent 95% CIs based on 5000 independent draws from the posterior distribution of parameters and imputed species wood durability. All predictors increased the probability of snag persistence except mean annual temperature, which decreased the probability of persistence. Other factors were examined but were not significant.

**Fig 3:**
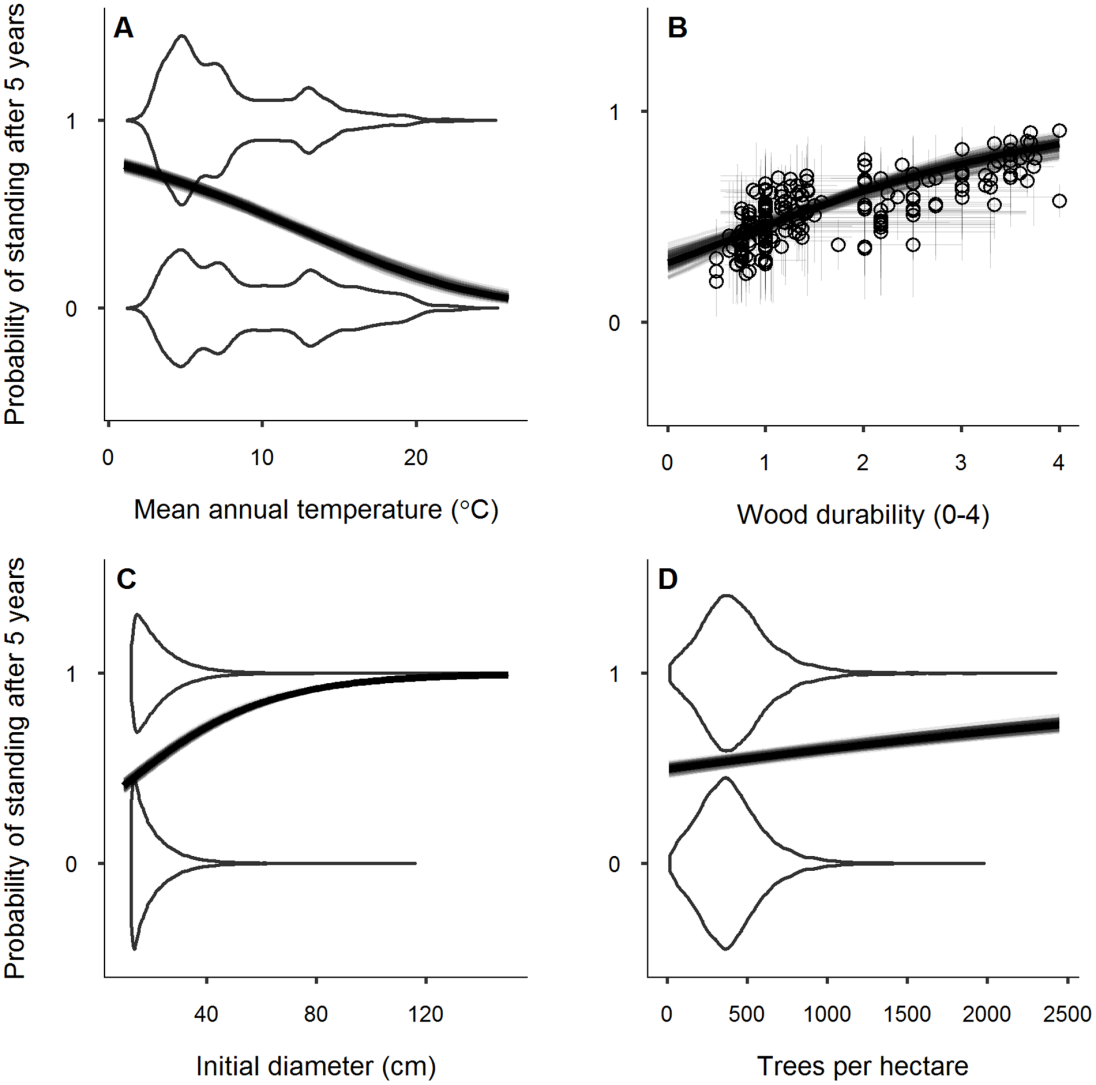
Increasing temperature reduces snag persistence, while species wood durability, diameter and stand density increase snag persistence. The thick central curves correspond to the mean of the posterior distribution for each effect, and the transparent curve overlay represents uncertainty by showing 100 curves drawn from the posterior distribution of the relevant parameters. The open circles in panel B correspond to the posterior means for all 205 species in the dataset with thin vertical bars representing 95% credibility intervals (CIs) for species-level effects and thin horizontal bars representing the 95% CI for imputed species-level wood durability. The vertical spread of the violin plots in panels A, C and D represent the distributions of the predictor values for observed standing (S = 1) versus fallen snags (S = 0).

Among many potential predictors, snag fall rates depended primarily on four factors, three of which control wood decay (Fig 2). The strongest predictor was average annual temperature (*AT*, Fig 2). Warmer forests had much faster rates of snag fall, even after controlling for spatial autocorrelation and species differences (Fig 3A). Assuming effects do not change with time, 2.4°C warming would reduce the annual probability of snag persistence by 0.035 (95% CI = [-0.083, -0.006]) with a corresponding decrease in expected half-life of 3.2 years (95% CI = [-12.5, -0.3]yr). Incorporating accelerated snag fall into a C model for a northern hardwood forest decreased both snag C and net ecosystem production (NEP, kg C ha^-1^ yr^-1^). Accelerated snag fall with 2.4°C warming reduced snag C by 1.85 Mg ha^-1^ (95% CI = [0.41, 3.31] Mg ha^-1^) or ~ 22%. The associated decrease in NEP was smaller, about 1% (95% CI = [0.5, 2.3]%) or 7 kg ha^-1^ yr^-1^. Both changes only reflected modelled differences in snag fall due to temperature, not potentially faster wood decay. The strong effect of temperature contrasted with the weak effect of average wind speed, a hypothesized predictor that was associated with slightly slower rates of snag fall in a subset of the data (S1 Fig).

After temperature, species wood durability (*DUR*) and individual tree diameter (*DIA*) had the next strongest effects (Fig 2). Snags from species with more durable wood stood significantly longer (Fig 3B). A recently dead tree with wood that is one unit less durable, all else being equal, reduced the annualized probability of snag persistence by 0.06 (95% CI = [-0.141, -0.01]) and the expected half-life by 4.5 years (95% CI = [-18.0, -0.4]yr). While wood durability strongly influenced rates of snag fall, the simplified models did not include a wood density effect, another hypothesized predictor. Likewise, snags with a wider diameter stood longer (Fig 3C). All else being equal, if two dead trees differed in diameter by 3.5 cm, which is the median growth expected to occur in the eastern US by 2050 (40), the larger tree had an annual persistence probability that was 0.017 higher (95% CI = [0.003, 0.044]) and an expected half-life 2.3 years longer (95% CI = [0.2, 9.1]yr).

Compared to other significant drivers of snag fall, stand density (*TPH*, trees per hectare) is uniquely associated with exposure to breaking forces and its effect on snag fall was much weaker (Fig 2). Nevertheless, a tree that died in a stand with 100 fewer trees per hectare had an annual persistence probability that was 0.005 lower (95% CI = [-0.013, -0.001]) and an average half-life that was 0.5 years shorter (95% CI = [-3.2, -0.1] yr) (Fig 3D). With the exception of the wood durability effect, the effect size of the three other important drivers (*AT*, *DIA* and *TPH*) diminished with advancing initial decay stage (Fig 2). We also examined whether slope or the average size of trees in the surrounding stand influenced snag fall rates, but like other variables expected to influence exposure to breaking forces, the effects of these factors were minimal.

**Fig 4:**
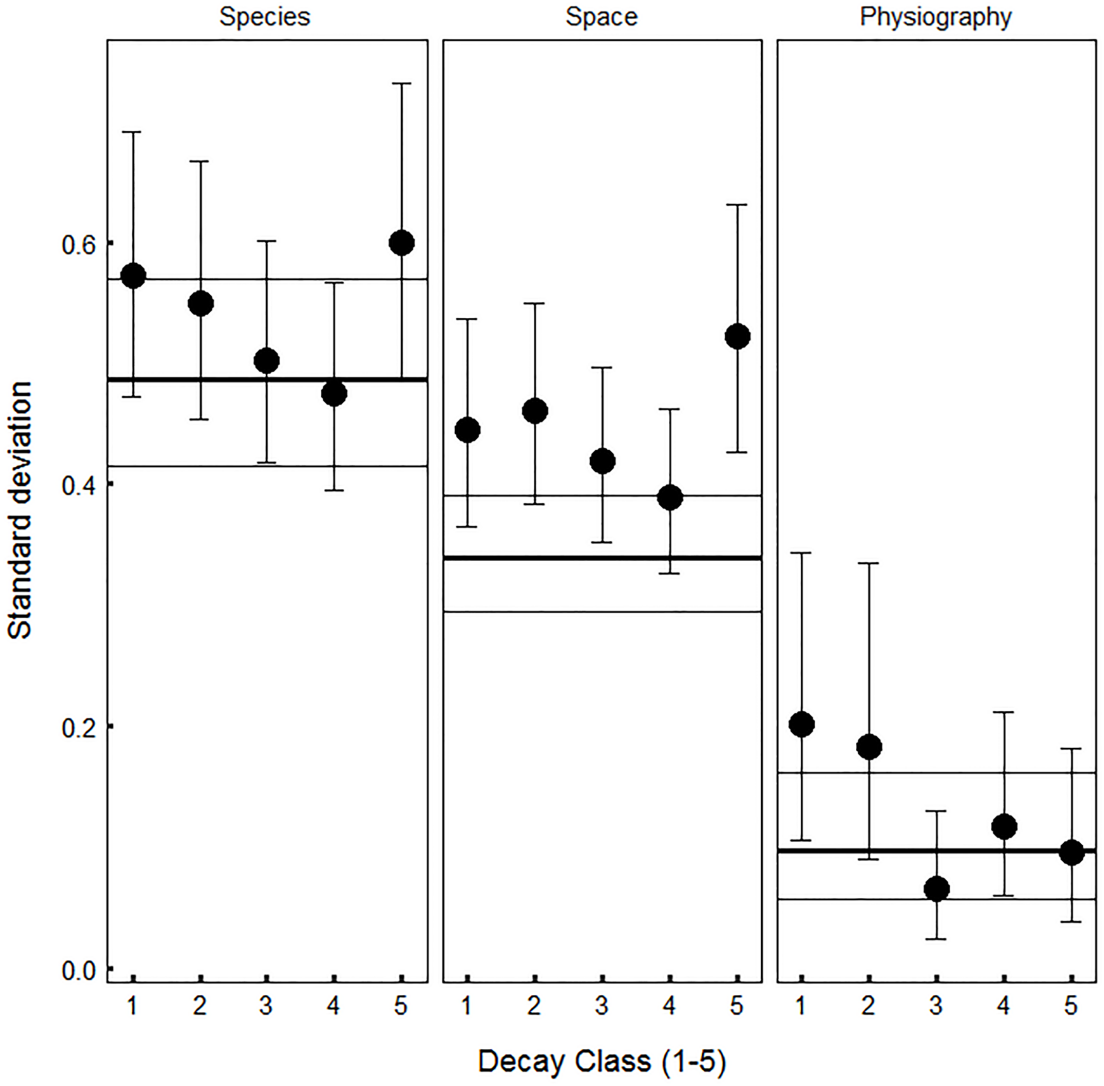
Residual species differences, spatial locations and physiographic classes have decreasing influence on variation in snag fall rates. Horizontal lines represent random effect standard deviations for all snags and filled circles represent random effect standard deviations for different progressive decay classes. Outer horizontal lines represent 95% credible intervals (CIs) for all snags. Thin vertical lines (whiskers) with filled circles represent 95% CIs based on 5000 independent draws from the posterior distribution of parameters. The standard deviations correspond to *σ*_*S*_ (species, Eq (5)), *σ*_*G*_ (space, Eq (4)), and *σ*_*P*_ (physiography, Eq (3)).

**Fig 5:**
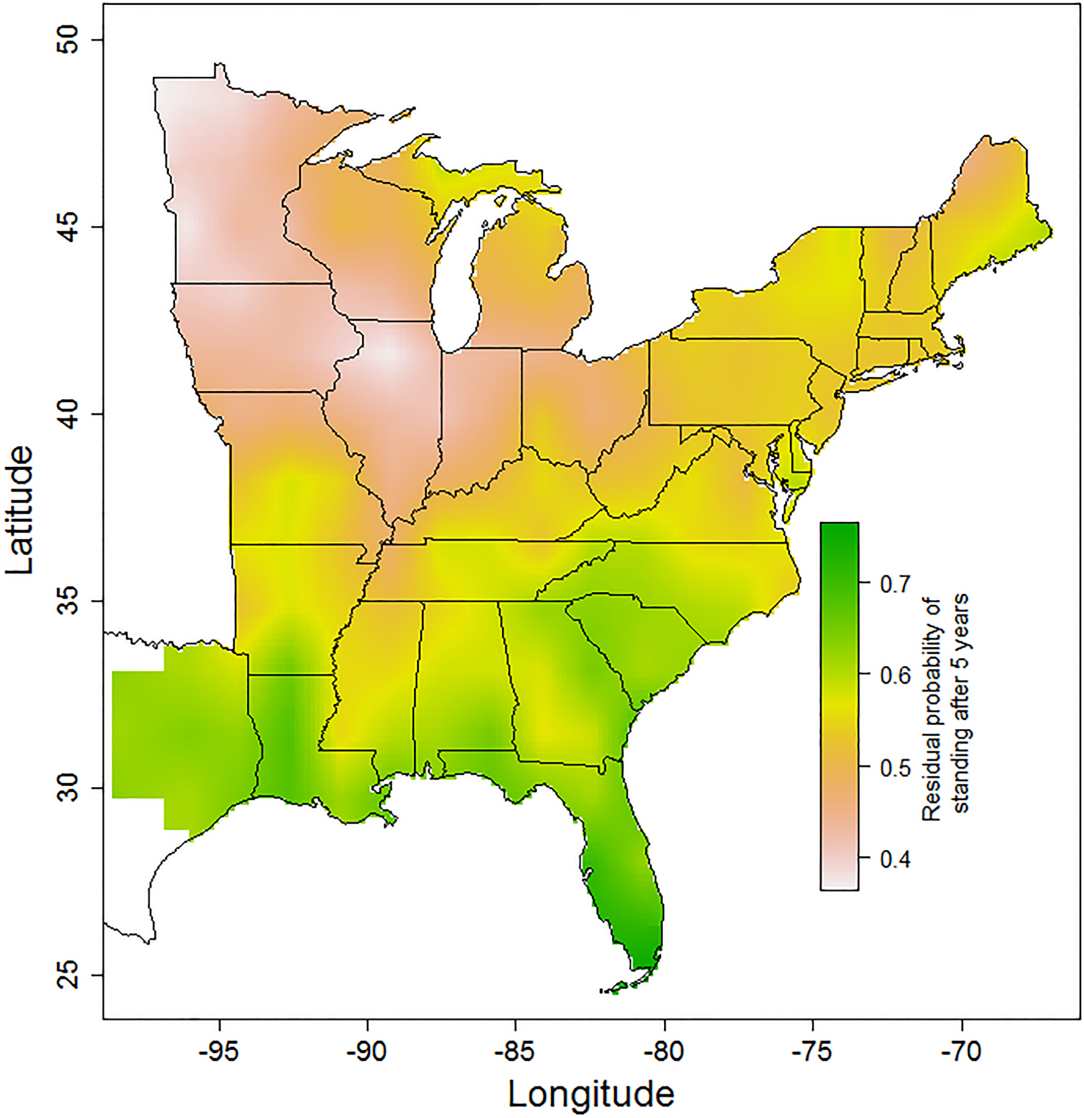
Snags in the upper Midwest have a higher residual probability of falling that snags in the southeasten United States. The surface is a bilinear interpolation over values estimated at the centers of 151 square (1.7° x 1.7°) grid cells. The bar at the right indicates the color scale denoting probability of snag persistence, with green colors representing slower than expected rates of snag fall after controlling for other drivers (predictor variables) that vary spatially, including tree species composition, stand density, physiographic class, and temperature.

Random effects associated with species differences, plot locations, and physiographic classes also influenced rates of snag fall. Snag persistence varied greatly among species (σ_S_ = 0.486, Fig 4), from an expected half-life of only 1.3 years (95% CI = [0.5, 3.1]yr) for *Tilia americana* (American Basswood) compared to 35.8 years (95% CI = [12.5, 90.8]yr) for *Maclura pomifera* (Osage Orange). Snag persistence also varied widely among different geographic locations (σ_G_ = 0.339, Fig 4). Controlling for variation in temperature, snags that occurred in the upper Midwest tended to fall at relatively fast rates (Fig 5). Compared to the considerable amount of variation explained by species and location, variation among physiographic classes was small (σ_P_ = 0.097, Fig 4), but these effects still indicate that snags occurring in habitats with loose or periodically disturbed soil are more likely to fall compared to those in habitats with standing water (S1 Table). Whether aggregated by tree species, spatial grid cell, or physiographic class, predicted snag persistence closely corresponded to observed snag persistence (S2, S3 and S4 Figs).

## Discussion

Global change drivers, from heat and drought to pests and intense storms, are accelerating mortality rates in Earth’s forests [40]. Shifts in tree demography, species composition, and carbon (C) cycling are all connected through the dynamics of standing dead trees [21]. What does a world with more new snags imply for critical ecosystem processes? Addressing this question requires predicting when snags fall by understanding why they fall. Our model accurately predicted whether or not two out of three snags fell during a decade-long national forest inventory involving repeated observations of almost 100,000 trees. Among many potential predictors, those that influenced wood decay played especially pivotal roles, raising the possibility of previously unrecognized feedbacks in the forest C cycle. A world where snags fall more quickly because they decompose faster is likely one where forest C residence times are shorter, further destabilizing climate. Moreover, quickly falling snags may degrade habitat for some threatened wildlife species while enhancing that of others, accelerate nutrient cycling and interact with important agents of forest change.

### Warm temperatures accelerate dead tree turnover

The most important driver of variation in snag fall rates, temperature, implies that climate change will dramatically alter deadwood structure and function. The strong effect of temperature on snag fall has seldom emerged from previous analyses, presumably because most have focused on snag dynamics in specific regions where temperature gradients are less dramatic [28–31,38,39]. At a continental scale, differences in overall snag fall rates between cooler Canadian forests (5.4% per year [11]) and warmer eastern United States forests (11.9% per year, this study) are consistent with a strong effect for temperature but confounded by differences in species composition and stand structure. After statistically controlling for the effects of other important drivers (such as species and wood properties), we substituted the effect of temperature across space for the effect of warming through time to predict that a model northern hardwood forest could lose more than 20% of snag C by mid-century. Considering that snags are a keystone structure for many endangered species, decreased snag lifespans could compound stresses faced by some threatened species [41], while simultaneously enhancing habitat for species that depend on recently fallen logs. Changes in species composition for deadwood associated organisms could affect forest biodiversity management plans. Moreover, snag fall rates are a major control on the timing of changes in net C balance following disturbance, including diebacks associated with increasing temperature [4,7,17]. We focused just on the effects of snag fall on the C cycle; however, warmer forests likely exhibit higher tree mortality, faster C emissions from both standing and down deadwood, and a faster transition from net C sinks to sources (Fig 1). Reduced C residence time in cooler forests that store C in snags today represents a quantifiable prediction for differences in C turnover among biomes [42,43].

The strong effect of temperature on snag fall contrasted with the weak effect of average wind speed, suggesting that variation in exposure to chronic mechanical stress poorly explains snag fall rates in eastern North America. This result contrasted with an analysis of drought-killed aspen (*Populus tremuloides*) in northwestern Canada, where snag fall rates were significantly higher in locations with faster wind speeds [28]. Snag sensitivity to wind throw may be species specific [44,45] or more strongly influenced by gusts. While we cannot directly quantify the effects of specific localized storms in our dataset, we note that blowdown events could contribute to the large variation in snag fall rates attributed to unmeasured spatially correlated processes. Specifically, elevated snag fall rates in the upper Midwestern United States could reflect the frequent incidence of strong convective storms in that region [46]. However, snag fall rates were not higher along the Gulf Coast where hurricanes generated a measurable influx of deadwood during the study interval [47]. Presumably, new down deadwood along the Gulf came directly from living trees that had blown over, raising the possibility of a distinct forest climate feedback involving stronger storms, higher mortality, and faster decay [17,48]. Living trees that fall because of blowdown, landslides, or other catastrophic events are likely to influence forest C cycling in important ways. More detailed analyses are necessary to identify what factors determine differences in blowdown rates of living and standing dead trees during storms.

### Wood durability accurately predicts snag fall

Snag dynamics also depended on species’ wood durability. Wood durability contrasts with more widely known wood mechanical properties, such as hardness and modulus of rupture, in that wood durability is only weakly correlated with wood density and measures long-term rather than initial wood strength [49]. For example, a common standard ranks species’ wood durability by comparing the useful lifespan of heartwood to that of sapwood from a weakly defended reference species [50]. Builders can use this information to predict the service lifespan of untreated outdoor wooden structures used as construction materials. In temperate settings, the difference between the first and second wood durability categories roughly corresponds to a 5-year difference in expected service lifetime [51]. In our model, the same difference in species wood durability corresponded to a 4.5-year difference in snag half-life. This close correspondence between the dynamics of buildings and standing dead trees highlights the ecological importance of this understudied wood functional trait. Larger wood durability datasets, involving more tree species and alternative ways of incorporating uncertainty between sources, may inform other important ecological phenomena.

The biological basis for variation in wood durability is largely unknown, but could reflect decay inhibition by secondary compounds that pigment heartwood and reduce wood permeability [36,49,52]. To the extent that wood durability corresponds to overall decay resistance, snags that fall quickly may decompose faster as logs [35]. Furthermore, wood durability might also predict resistance to internal decay in living trees, identifying species that are likely to be hollow and more prone to falling while alive [53]. For both of these reasons, species with less durable wood could be targets for interventions to enhance C storage and manage risks of hazard tree failure. Even species that produce durable wood currently may produce less decay resistant wood in the future. CO_2_ fertilization tends to accelerate tree growth, and fast growing trees tend to produce less durable wood [34,35]. In a recent wood decay experiment involving both plantation-raised and old growth scots pine, less durable wood decomposed more quickly across a range of temperatures and fungal strains [37]. Faster growth with higher CO_2_ raises the possibility that trees may produce less durable wood, so that they decay and fall more quickly (Fig 1). Lower concentrations of decay-limiting extractives in more recently deposited heartwood would support this conclusion.

While wood durability was an important predictor of snag fall, the large residual interspecific variation (Fig 4) indicates that other unmeasured species traits also play important roles determining whether snags break or uproot. For example, in a tropical dry forest, interspecific differences in tree fall rates were associated with tree height, rooting depth, and wood density [44]. Surprisingly, intact wood density was not a significant predictor for snag fall in eastern North American forests, indicating that standing dead tree failure depends less on initial wood mechanical strength and more on how strength changes with decay [19]. However, the range of wood density in the temperate zone is much narrower than what occurs in the tropics [52], raising the possibility that tropical forests show more variation in snag fall rates driven by increased variation in wood density.

### Effects associated with breaking forces weaken with decay

Significant effects for stem diameter and stand density indicate that exposure to breaking forces also influences snag dynamics. Large stem diameters, which can slow wood decay and increase mechanical resistance to breaking forces [9], were associated with slower rates of snag fall. The slow fall rate for larger snags may moderate some effects of climate change on forest C cycling. Large trees store more C [54] and may be more sensitive to mortality during drought [55]. Yet, as dead trees, they tend to stand longer, providing more time for proactive management strategies like salvage harvest while their wood remains intact. Large, relatively persistent snags may also preserve habitat for some important wildlife species [1].

While tree size influences both wood decay and toughness, stand density more directly affects exposure to breaking forces. We found that snags in denser stands showed slightly slower falling rates. This result is consistent with the expectation that dense stands shield snags from breaking forces and could indicate that future forests with lower stem densities may have higher rates of snag fall. However, in dense forests affected by dieback, the protective effect of stand density could be offset by accelerated decay with increasing abundance of wood decay fungi and wood boring beetles [28] and the possibility for a domino effect [27]. Nevertheless, the effect of stand density, like the effects of diameter and temperature, declined with advancing decay class, which suggests that wood decay eventually overrides the effects of other factors.

### Wood decay, forest disturbances, and distinctive snag legacies

Ultimately, integrating snag dynamics into risk projections for changing forests requires understanding how deadwood decay and toughness vary with different mechanisms of tree mortality. Harvest, drought, pest outbreaks, and fires promoted forest C loss at the same order of magnitude as fossil fuel consumption in western North America [56]. While the relative roles of drivers vary, harvest is the biggest single driver of global forest C loss [57]. In northern forests, experimental harvests raised soil temperatures, driving faster wood decay [58] and accelerated fall rates for both naturally recruited and artificial snags [32]. Both experimental results in managed forests are consistent with our analysis of snag dynamics in unmanaged forests across the eastern United States, where increased temperature and decreased stand density both accelerated tree fall. Whatever standing deadwood remains in managed locations is likely to fall and decay more quickly. Identifying whether these connections extend to recreational or urban forests has major implications for mitigating hazard tree risks.

While faster wood decay and snag fall might accelerate C emissions after harvest, forest dieback during drought may influence snag dynamics differently. Wood decay is strongly moisture limited [9]. Consequently, prolonged drought is likely to slow wood decay and snag fall. While drought should slow wood decay and snag fall, in some cases, drought is not always the proximate cause for tree mortality. Often, drought and heat stress interact to trigger pest outbreaks or fires [42]. These change agents can have distinctive impacts on snag fall and C dynamics [4,59]. Wood boring beetles can structurally compromise snags and spread wood decaying microorganisms, increasing rates of snag fall [60]. Fire may play an even more complicated role. Intense forest fires partially consume existing snags while creating new snags by killing trees. Charred snags have different exposure to breaking forces and decay resistance. In California, snags in burned stands fell faster due to a combination of structural weakening and increased exposure to wind [31]. Both of these results are consistent with our analyses of snag dynamics in unburned stands in the eastern United States. While burning is likely to reduce snag C, residual charred wood decays extremely slowly, which can enhance stabilized soil C depending on fire return intervals. In Oregon, reburned stands showed persistent differences in deadwood structure [39] although fire return interval had relatively little impact on snag abundance during an experiment in southern Florida [61].

Overall, warm temperatures and faster wood decay accelerate standing dead tree fall. Associated reductions in snag C residence time may increase the sensitivity of forests to climate forcing, especially if higher CO_2_ reduces tree species wood durability. Forests with fewer snags and more logs may have different habitat value for deadwood-dependent species. Where people use forests, risks associated with snag fall may change as well. Improving forest resource management will require a better understanding of how snag fall rates interact with other agents of forest disturbance, especially strong storms and fires, as well as a thorough examination of potential connections between the drivers of snag fall in unmanaged forests with living tree fall rates including those in urbanized areas.

## Materials and methods

### Response variable: Snag fall

We evaluated the persistence of standing dead trees in permanent forest inventory plots resurveyed by the USFS Forest Inventory and Analysis (FIA) Program [62]. In 1999, FIA began recording the presence of snags, defined as main boles of stems at least 12.7 cm in diameter at 1.37 m height (diameter at breast height or DBH) and leaning <45° from vertical. Annual resurveys of 20% of the FIA plots yield an average resurvey interval of 5 years. We queried the FIA database for all resurveyed and unmanaged plots that included one or more snags during the initial survey. Standing dead trees are tracked through time by the FIA program until they no longer meet their associated definition (fallen or decayed/fractured below minimum size thresholds). Given the importance of identifying the distinctly different live and dead tree populations across the US for national resource assessments, care is given to accurately identify trees as they transition from live, to dead, to down dead via the field measurement variables of tree status and reconciliation codes [63]. As such, when the FIA field records identified individual snags as no longer qualifying as a standing dead tree on plots where there were no recent forest management activities (i.e., logging equipment knocking over dead trees), our study considered those snags as fallen. We limited our analysis to plots that had been revisited twice between 2000, when the new nationally consistent criteria for snag inclusion were adopted, and 2010, when most plots had been resurveyed.

### Predictors of snag fall

We estimated climatic conditions at the reported location for each plot, which, for privacy reasons, is within 1 km of the actual plot location. We estimated average wind speed (*WND*) at 10 m altitude during the interval from 2000–2010 based on the National Atmospheric North American Regional Reanalaysis [64], which provides data at a resolution of approximately 32 km^2^. We estimated average annual temperature (*AT*) over the 30-year interval from 1981 to 2010 based on PRISM, which estimates temperatures at a resolution of 800 m^2^ [65].

For quantifying effects of factors that vary at the landscape, stand, and individual scales, we obtained additional covariates based on measurements taken during the FIA surveys. For topography, we included subplot slope (*SL*) and physiographic class. FIA defines physiographic class using a cross classification of forest habitats by hydrology and landform (S1 Table). At the stand scale, we included both average size and density of living trees. For an estimate of average size, we calculated quadratic mean diameter (*QMD*) of all living trees over 12.7 cm DBH. To estimate density, we calculated the number of living trees per hectare (*TPH*) over 12.7 cm DBH. At the scale of each individual snag, we included both DBH (*DIA*) and initial decay class (*DC*). FIA assigns snags to one of five progressive decay classes based on external conditions [62].

To quantify species-level variation in wood traits, we compiled two additional datasets. We drew our estimates of species-level intact wood density (*DEN*) from Miles & Smith [66]. We also compiled information on tree species wood durability (*DUR* S1 Dataset). Wood durability describes how untreated wood resists degradation by microbes and insects using an ordinal scale. This scale has a direct quantitative interpretation under certain materials standards. For example, the European standard [50] compares the lifespan of heartwood stakes to that of *Pinus sylvestris* sapwood. Sapwood stakes from *Pinus sylvestris* resist impact breakage tests for about 5 years when suspended above ground in temperate conditions [51] defining the lowest reference class, “Not durable”. The next class, “Slightly durable” lasts between 1.2 and 2x longer, “Moderately durable” wood lasts between 2 and 3x longer, “Durable” wood lasts between 3 and 5x longer, and “Very durable” wood lasts more than 5x longer. This standard is similar to the discontinued United States standard [67]. We compiled descriptions of tree species wood durability from several sources including the Forest Products Lab Wood Handbook [34] and Wood Properties Techsheets [68], the Natural Durability of Wood : a Worldwide Checklist of Species [49], the American Hardwood Export Council Guide to Species [69], the Wood Database [70], the USDA Fire Effects Information System [71], the Forest Trees of Florida Handbook [72] and the Utah State University Tree Browser [73]. For maximum consistency with the materials standards terminology, we defined the least durable classification within a source (e.g. “not durable”, “non-resistant”) as durability 0, the next category (e.g. “slightly durable to perishable”) as durability 1, the next highest (e.g. “moderately durable”) as durability 2, the second highest (e.g. “durable” or “resistant”) to durability 3 and the highest category (e.g. “very durable” or “very resistant”) to durability 4. For species described using a range of descriptors within a source (e.g. moderately to very durable), we scored them to intermediate categories (i.e. durability 3). We resolved variation among sources by taking the arithmetic mean for each species with more than one estimate.

### Model structure

Our goal was to identify the factors that most strongly influence the rate at which snags fall. We did so by estimating the probability, *p*_*ijkl*_, that any individual snag *i* (*i* = 1, 2, …, *n =* 99,213), remains standing (*S*_*ijkl*_ = 0 or 1) at the end of a census interval of length *t* (years) in a plot with a specified physiographic class *j* (*j* = 1, 2, …, *m* = 16), spatial location *k* (*k* = 1, 2, …, *p* = 151), and that had been previously identified as species *l* (*l* = 1, 2, …, *q* = 205). We assumed *S*_*ijkl*_ represents a single Bernoulli trial:

$$S_{ijkl}\sim Bernoulli({p_{ijkl}}^{t})$$ (1)

We then analyzed the annualized probability of standing *p*_*ijkl*_ as a linear function of covariates in a logistic regression framework:

$$logit(p_{ijkl})=log\left(\frac{p_{ijkl}}{1-p_{ijkl}}\right)=\beta_{o}+\;\sum t_{ijkl}\rho +u_{j}+v_{k}\;+w_{l}$$ (2)

**t**_*ijkl*_ is a row vector of continuous predictors and ***ρ*** is a vector of associated effects. We covariate-centered the continuous predictors (**t**’s) by subtracting their mean such that *β*_*o*_ is the annualized baseline log odds of remaining standing at “average” covariate conditions. We evaluated many candidate models including effects for tree *DBH*, stand *QMD* and *TPH*, plot *SL*, *WND* and *AT* (S1 Appendix). In addition to continuous covariate effects, *u*_*j*_ is the effect of physiographic class *j*, *v*_*k*_ is the effect of location *k*, and *w*_*l*_ is the effect of species *l*.

To account for variation among physiographic classes, we modelled their effects as coming from a population of potential effects:

$$u_{j}\sim Normal(0,\sigma_{P}^{2})$$ (3)

The effects (*u*_*j*_’s) are constrained to sum to zero relative to the baseline persistence rate, *β*_*o*_, and $\sigma_{P}^{2}$ is the variance among physiographic class effects.

To model the spatially structured location effects, we assigned each location to a 1.7° x 1.7° grid consisting of *k* = 1, 2, …, *p* = 151 cells. This grid resolution was small enough to capture coarse spatial variation while being sufficiently large to include multiple plots in most cells. We then estimated spatially structured effects as a Gaussian conditional autoregressive process [74], such that for grid cell *k* ≠ *h*:

$$v_{k}|v_{h}\sim Normal\left(\frac{\sum_{h\in \delta_{k}}a_{kh}v_{h}}{a_{k+}},\frac{1}{a_{k+}{\;\sigma }_{G}^{2}}\right)$$ (4)

*δ*_*k*_ defines the cells that are spatially adjacent to the focal cell *k*, *a*_*kh*_ is a binary operator that equals 1 only if cell *h* is adjacent to *k*, *a*_*k*+_ is the number of cells adjoining *k*, and ${\;\sigma }_{G}^{2}$ is the variance of the conditional Gaussian distribution. As with our estimates of physiographic class effects, we constrained these effects (*v*_*k*_’s) to sum to zero so that the spatial autoregressive effects represent local deviations from the baseline persistence rate, *β*_*o*_.

For modelling species-level effects, we incorporated known differences in species’ wood traits using a hierarchical approach:

$$w_{l}\sim Normal(\mu_{l},\sigma_{S}^{2})$$ (5)

*μ*_*l*_ is the expected species-specific deviation from the baseline persistence probability, *β*_*o*_, and $\sigma_{S}^{2}$ is the variance that describes variability among species. We constrained the species effects (*w*_*l*_’s) to sum to zero and modeled the expected species-specific deviations (*μ*_*l*_) as a function of wood durability (*DUR*):

$$\mu_{l}=\beta_{DUR}*\;\left(DUR_{l}-\bar{DUR}\right)$$ (6)

*β*_*DUR*_ *is* the effect of wood durability on the probability of snag persistence, and $\bar{DUR}$ is the average wood durability index, computed across all species for which *DUR* estimates were available.

Several species lacked *DUR* estimates. Rather than exclude them, we imputed their *DUR* values based on *DUR* estimates available for other species by assuming a hierarchical model for *DUR* based on a coarse taxonomy [33] whereby species are nested in family:

$${logit\;(DUR}_{l}/4)\;\sim \;Normal(\mu_{f(l)},\sigma_{f}^{2})$$ (7)

Where the logit transformation (see Eq 2) and rescaling projects the [0–4] interval-constrained *DUR* variable to the real number line; *f*(*l*) denotes family *f* associated with species *l*;*μ*_*f*_ is the family-level logit-transformed *DUR* value; and $\sigma_{f}^{2}$ is the variance that describes variability in logit-transformed *DUR* among species within families. Then we modeled the family-level means as nested in division (i.e. gymnosperm versus angiosperm):

$$\mu_{f}\sim Normal(\mu_{d(f)},\sigma_{d}^{2})$$ (8)

*d*(*f*) denotes division *d* associated with family *f*; *μ*_*d*_ is the logit-transformed *DUR* for either angiosperms or gymnosperms; and $\sigma_{d}^{2}$ is the variance that describes variability in logit-transformed *DUR* among families within divisions.

### Model implementation and adequacy

We fit the above model in a Bayesian framework, with diffuse normal priors, *Normal*(0,1000), for all of the continuous effect parameters (i.e., each element of ***ρ*** in Eq (2) and *β*_*DUR*_ in Eq 6) as well as *μ*_*d*_ in Eq (8). For the baseline fall rate parameter, *β*_*o*_, we used a flat prior. We specified broad uniform priors, *Uniform*(0,100), for the standard deviation terms associated with each variance component (i.e. *σ*_*P*_, *σ*_*S*_, *σ*_*f*_, *σ*_*d*_). Finally, following [46], we specified a *Gamma*(0.5, 2) prior for the precision of the conditional Gaussian distribution for the spatially structured effects (i.e. $\sigma_{G}^{-2}$, Eq (4)). Our use of minimally informative priors allows the data to largely inform the posterior distribution. Furthermore, no other studies have employed Bayesian techniques to estimate snag fall rates so informative prior distributions are lacking.

We implemented the above model using OpenBUGS, V3.2.1 rev 781 [75] (S2 Appendix). OpenBUGS employs Markov Chain Monte Carlo sampling for model parameters from the joint posterior distribution. We ran five parallel chains for at least 110000 iterations per chain. We assessed convergence of the chains to the posterior when the upper limit of the 95% CI Brooks-Gelman-Rubin (BGR) statistic [76] fell below 1.05. We then discarded the first 10000 samples as burn-in and thinned each chain by a factor of 40 to reduce within-chain autocorrelation. We repeated the process for the full dataset and for subsets of the original dataset representing each decay class (S1 Appendix).

We assessed model adequacy by comparing observed to expected snag persistence given the hierarchical model and posterior mean parameter estimates. For every observation *S*_*ijkl*_, we rounded model-based expected values for *p*_*ijkl*_^*t*^ to either 0 or 1. Then we calculated both the proportion of correct predictions of *S*_*ijkl*_ and the 95% confidence interval for the odds ratio of observed versus predicted values using the function “fisher.test” in R v 3.2.5 (package “stats”). We also plotted the expected versus observed counts at every level of the three categorical predictors (species, spatial grid and physiographic class).

### Parameter interpretation

For continuous predictors (e.g., **ρ** in Eq (2) or *β*_*DUR*_ in Eq (6)), we computed a standardized effect size by dividing the posterior mean and 95% CI limits by the standard deviation of the corresponding covariate data. This provides an estimate for the relative effect of a standard change in the predictor near the mean of the data on the logit scale. Because we imputed wood durability (*DUR*) for some species, our estimate of the standardized effect size for this variable was averaged over 5000 draws from the posterior distribution for the missing *DUR* estimates. For the relative influence of categorical predictors, we also report the posterior mean and 95% CI for the standard deviation of the random effects (i.e., *σ*_*P*_, *σ*_*S*_, and *σ*_*G*_).

To relate the aforementioned effects to expected changes in rates of snag fall, we computed average predictive comparisons [77,78]. This approach allows us to compare the difference (*Δ*) in the response of interest (*y*)—which is related to different indices of rates of snag persistence—with a specified change (ξ) in a predictor variable of interest (*x*) as:

$$\Delta (\xi ,\;t,x,X)\;=\;\frac{\sum_{r\;=\;1}^{n}\left(E(y|x_{r}+\xi ,\;\theta ,X_{r})-E(y|x_{r},\;\theta ,X_{r})\right)}{n}$$ (9)

*r* denotes an individual tree, as indexed by all observed unique combinations of *i*, *j*, *k*, and *l* (see Eq 1); **θ** represents a vector of estimated parameters (i.e., includes ***ρ***, *β*_*o*_, *β*_*DUR*_, and all σ terms); and **X** is a matrix of all other predictors excluding the focal predictor, *x* (**X** and *x* involve different combinations of **t** in Eq (1) and *DUR* in Eq (6)). The specified values for ξ for each variable were chosen as reasonable estimates for the direction and magnitude of change in eastern North American forests by mid-century. For mean annual temperature in 2050, we used the estimated 2.4°C increase from the CMIP3 model projection for the RCP8.5 emissions scenario [79]. For diameter (DBH), we used the mean diameter growth for eastern North American forests in a 35 year interval as estimated by [80] to infer the associated change in DBH for a “typical” tree. For mid-century TPH, we assumed that most forests would lose trees due to stand thinning and mortality. We note that the proposed values for ξ are simply to put effects on a biologically relevant scale and that they are considered one at a time, in an additive fashion.

We expressed the difference in expected rates of snag persistence in two different ways. First we computed the average predictive difference in the annualized probability of remaining standing such that:

E(y|x,θ,X)=Pr(S|x,θ,X)=p(x,θ,X) (10)

where *p* is the annualized probability of a snag persisting, which is predicted from Eq (2). Then, for snags in the earliest DC, we also computed the average predictive difference in half-life in years as:

$$T_{1/2}\;=\;\frac{log(0.5)}{log(Pr(S|\;x,\theta ,X))}$$ (11)

where *Pr*(*S*|*x*,**θ**,**X**) is described in Eq (10). To incorporate parameter uncertainty, we report distributions for these average predictive differences based on 5000 draws from the posterior distributions of the parameters.

Finally, we examined the effects of projected changes in snag persistence on forest structure and function in a simple, spatially implicit, discrete time forest C model [81]. We modified the model by specifying separate detritus pools for litter, snags, and logs. We then parameterized the model to approximate the C budget of a well-characterized north temperate hardwood forest, Hubbard Brook Experimental Forest, using internal transitions estimated by [82], along with snag persistence times estimated from our model, and decay rates for litter, snags, and logs based on literature information [9,83,84]. We evaluated steady state snag C and maximum net ecosystem productivity (NEP) under contemporary conditions relative to those projected for 2050 based only on a change in snag persistence time due to projected temperature change. This approach estimates the sensitivity of snag C and NEP to snag fall rates *per se* without including changes in decay rate or potential feedbacks mediated by climate (e.g. Fig 1). We assessed uncertainty in our sensitivity estimate by repeating the simulation for 1000 parameter estimates drawn from the distribution of predictive comparisons. Model code is available in S3 Appendix.

## Supporting information

S1 Appendix Detailed description of model simplification procedure. (DOCX)

S2 Appendix BUGS language code for the simplified snag fall model. (DOCX)

S3 Appendix R code for the simple forest C model. (DOCX)

S1 Fig Snags of intermediate decay class are less likely to fall in locations with faster average wind speeds. Thick central curve corresponds to the posterior mean for the effect of average wind speed at 10m on decay class 2 snag persistence and the transparent curve overlay represents uncertainty by showing 100 curves drawn from the posterior distribution of the relevant parameters. The vertical spread of the violin plots represents the distributions of the predictor values for standing (S = 1) versus fallen trees (S = 0).
(TIFF)

S2 Fig The predicted proportion of snags standing closely corresponds to the observed proportion of snags standing when aggregated by 205 species. Symbol diameter is scaled by species abundance.
(PDF)

S3 Fig The predicted proportion of snags standing closely corresponds to the observed proportion of snags standing when aggregated within 151 1.7°x1.7° spatial grid cells. Symbol diameter is scaled by the abundance of snags per grid cell.
(PDF)

S4 Fig The predicted proportion of snags standing closely corresponds to the observed proportion of snags standing when aggregated within 16 physiographic classes. Symbol diameter is scaled by the abundance of snags per physiographic class.
(PDF)

S1 Table Snags occurring in physiographic settings with loose or disturbed soils are significantly more likely to fall compared to snags in settings with standing water. The final column indicates subsets of the data where the 95% CI for the effect excluded 0.
(DOCX)

S2 Table Model selection criteria applied to all first-order non-hierarchical models for snag persistence in every subset of the data. The set of parameters selected via backwards elimination from the fully hierarchical model is indicated in bold.
(XLSX)

S1 Dataset Compilation of wood durability estimates for species in the eastern United States drawn from various sources. The least resistant category was set as 0 and the most resistant category as 4. Sources that used ambiguous designations (e.g. non-resistant or slightly resistant) were given the average numeric score of the corresponding categories (i.e. 0.5).
(XLSX)
