# Supplementary material for: When a tree falls: Controls on wood decay predict standing dead tree fall and new risks in changing forests
Source: PLoS One. 2018 May 9;13(5):e0196712. doi: 10.1371/journal.pone.0196712 (PMC5942820; doi:10.1371/journal.pone.0196712)
Supplement: S1 Appendix — (DOCX) [file pone.0196712.s001.docx]

**Appendix S1:** Model simplification procedure.

Equations (1)-(8), describe the final model used for inferences presented in the Results. To arrive at this model, we began with models that included all continuous predictors: *DIA, SL, QMD, TPH, AT* and *WND* estimated at the snag-level as well as *DEN* and *DUR* estimated at the species level*.* We fit these models in a Bayesian framework using the same priors and MCMC sampling approach described in the Methods. We simplified models using a backwards elimination approach. For example, if the 95% credible interval (CI) for a particular covariate effect (such as one component in **ρ**) included zero, we eliminated the corresponding variable from the model. We repeated this procedure for the full dataset and for subsets of data representing each individual DC.

As a validation of the backwards elimination process, we also compared similar, maximum likelihood-based generalized linear models (GLMs) to our Bayesian results. We fit GLMs using the R v. 3.2.5 [1] “glm” function in the “lme4” package [2] using a custom link function developed by B. Bolker (<https://github.com/bbolker/bbmisc/blob/master/logexp/logexp.rmd>) that accommodates variation in resurvey interval length in logistic regression by raising the estimated probabilities to the exponent *t* as in equation (2). Because glm and lme4 cannot accommodate multiple random or hierarchical effects, each of our models included fixed effects for all categorical predictors. We then compared the set of all possible first order GLMs for all combinations of continuous predictors $\mathbf{t}_{ijkl}$ using the Akaike Information Criterion (AIC, 3) (see Table S2). In each case, the model selected by backwards elimination in a Bayesian context was within 3 log-likelihood units of the GLM selected based on AIC, indicating that both procedures converged on similar sets of predictors.

After backwards elimination and validation using maximum likelihood, we reran the simplified hierarchical models using the same MCMC settings and convergence criteria that we applied to the full model, except that we extended the MCMC chains for some slowly mixing parameters until the autocorrelation-adjusted effective sample size was at least 1000.

*Decay class effects:* Our preliminary analyses indicated that many of the effects varied with the DC of individual snags. Unexpectedly, resurvey crews assigned the same snag to a less advanced class in 15.9% of all repeated observations. The relatively high frequency of apparent DCs reversals prevented us from estimating a full model for expected snag lifetimes based on transition probabilities between DCs [4,5]. Rather, we analyzed how initial DC interacts with other factors via two approaches. First, we estimated all of the parameters in equations (1) – (8) for all snags, irrespective of DC. This approach estimates the influence of predictors for a snag of unknown degree of decay. Second, we estimated all of the parameters for each of the five initial DCs; this was accomplished by implementing equations (1) – (8) separately for subsets of data corresponding to the five unique initial DCs. Compared to the first approach, the set of parameters estimated for each DC represents a full interaction model where every parameter is allowed to vary by DC [6].

***References***

1. R Development Core Team. R: A Language and Environment for Statistical Computing [Internet]. Vienna, Austria; 2010. Available from: http://www.r-project.org

2. Bates D, Maechler M, Bolker B, Walker S. lme4: Linear mixed-effects models using Eigen and S4. R package version 1.1-7, http://CRAN.R-project.org/package=lme4. R Packag version [Internet]. 2014; Available from: http://scholar.google.com/scholar?hl=en&btnG=Search&q=intitle:Linear+mixed-effects+models+using+Eigen+and+S4#0

3. Akaike H. Information theory and an extension of the maximum likelihood principle. In: Petran BN, Csaki F, editors. International Symposium on Information Theory. Akademiai Kiadi; 1973. p. 267–81.

4. Vanderwel MC, Caspersen JP, Woods ME. Snag dynamics in partially harvested and unmanaged northern hardwood forests. Can J For Res. 2006;36(11):2769–79.

5. Aakala T, Kuuluvainen T, Gauthier S, De Grandpré L. Standing dead trees and their decay-class dynamics in the northeastern boreal old-growth forests of Quebec. For Ecol Manage. 2008;255(3–4):410–20.

6. Gelman A, Hill J. Data analysis using regression and multilevel/hierarchical models. Policy Analysis. 2007. 1-651 p.
