# Supplementary material for: When a tree falls: Controls on wood decay predict standing dead tree fall and new risks in changing forests
Source: PLoS One. 2018 May 9;13(5):e0196712. doi: 10.1371/journal.pone.0196712 (PMC5942820; doi:10.1371/journal.pone.0196712)
Supplement: S2 Appendix — (DOCX) [file pone.0196712.s002.docx]

#this is the reduced model used for inference, not the full model used to explore every parameter

model{

for (i in 1:n.TREE){ #Tree-level loop

STAND[i]~dbern(s[i]) #s[i] is probability of standing during the observed interval

s[i]<-pow(p[i],YR[i]) #p[i] is the annualized probability of standing

logit(p[i])<-beta.0 + (DIA[i]-mean.DIA)*beta.DIA #Tree level covariates for DBH (DIA:cm)

+ (TPH[i]-mean.TPH)*beta.TPH + (AT[i]-mean.AT)*beta.AT #plot level covariates for quadratic mean diameter (QMD: m ns.), trees per hectare (TPH) average annual temperature (MAT), average windspeed (WND ns)

+ beta.PHY[phy[i]] + beta.GRID[grid[i]] + beta.SPP[spp[i]] #random effects for physiographic class (PHY: 16 categories) spatial location (GRID: 151 1.7 degree cells) and species (SPP: 205)

}

beta.GRID[1:n.GRID]~car.normal(adj[],weights[],num[],tau.GRID) #The beta.GRID as a conditional autoregressive process

for (j in 1:n.PHY){ #PHY R.E. loop with sweeping for identifiability

beta.PHY[j]~dnorm(0,tau.PHY)

beta.PHY.adj[j]<-beta.PHY[j]-mean.beta.PHY

}

for (k in 1:n.SPP){ #SPP regression with means for species effects including traits

beta.SPP[k]~dnorm(mu.SPP[k],tau.SPP)

mu.SPP[k]<-beta.0.SPP+beta.DUR*(DUR.cut[k]-mean.DUR.cut)#species-level regression

beta.SPP.adj[k]<-beta.SPP[k]-mean.beta.SPP #adjusted species effects including trait effects

l.DUR[k]~dnorm(mu.fam[fam[k]],tau.fam) #the durability data are read in on the logit scale

DUR[k]<-exp(l.DUR[k])/(1+exp(l.DUR[k]))*4 #transforming the logit data to the original scale

DUR.cut[k]<-cut(DUR[k])

}

for(l in 1:n.FAM){ #taxonomic data imputation for wood durability over taxonomic families and divisions. These levels provided the best fit as per DIC.

mu.fam[l]~dnorm(mu.div[div[l]],tau.div)

}

#mean centered predictors

mean.DIA<-mean(DIA[])

mean.AT<-mean(AT[])

mean.DUR.cut<-mean(DUR.cut[])

mean.TPH<-mean(TPH[])

#sweeping non-identified REs into adjusted intercept for improved sampling

mean.beta.PHY<-mean(beta.PHY[])

mean.beta.SPP<-mean(beta.SPP[])

beta.0.adj <- beta.0 + mean.beta.PHY + beta.0.SPP

#priors

beta.0~dflat()

beta.AT~dnorm(0,0.001)

beta.DIA~dnorm(0,0.001)

beta.DUR~dnorm(0,0.001)

beta.TPH~dnorm(0,0.001)

beta.0.SPP~dnorm(0,0.001)

mu.div[1]~dnorm(0,0.001)

mu.div[2]~dnorm(0,0.001)

#precisions

tau.PHY<-pow(sigma.PHY,-2)

sigma.PHY~dunif(0,100)

tau.SPP<-pow(sigma.SPP,-2)

sigma.SPP~dunif(0,100)

tau.GRID~dgamma(0.5,2)

sigma.GRID<-sqrt(1/tau.GRID)

tau.fam<-pow(sigma.fam,-2)

sigma.fam~dunif(0,100)

tau.div<-pow(sigma.div,-2)

sigma.div~dunif(0,100)

ignore<-WND[1]+DEN[1]+SL[1]+QMD[1]

}
