# Supplementary material for: When a tree falls: Controls on wood decay predict standing dead tree fall and new risks in changing forests
Source: PLoS One. 2018 May 9;13(5):e0196712. doi: 10.1371/journal.pone.0196712 (PMC5942820; doi:10.1371/journal.pone.0196712)
Supplement: S3 Appendix — (DOCX) [file pone.0196712.s003.docx]

#parameters

Lmax<-146.6

B1<-0.02

B2<-2

sn.m<-0.00678349249658936

sn.k<-0.0045

sn.f<-0.151

lg.k<-0.045

lt.m<-0.0176162346521146

lt.k<-0.5

sk<-0.009

sf<-0.0105701754385965

#model adapted from Harmon 2007

yr<-0:300

#stocks

living<-Lmax*(1-exp(-B1*yr))^(B2)

litter<-c(0)

for (i in 2:301){

lt<-litter[i-1]*(1-sf-lt.k)+lt.m*living[i]

litter<-append(litter,lt,after=length(litter))

}

snag<-c(0)

for (i in 2:301){

sn<-snag[i-1]*(1-sn.f-sn.k)+sn.m*living[i]

snag<-append(snag,sn,after=length(snag))

}

log<-c(0,0)

for (i in 3:301){

lg<-log[i-1]*(1-sf-lg.k)+sn.f*snag[i-1]

log<-append(log,lg,after=length(log))

}

soil<-c(0,0)

for (i in 3:301){

s<-soil[i-1]*(1-sk)+(log[i-1]+litter[i-1])*sf

soil<-append(soil,s,after=length(soil))

}

C2005<-data.frame(living,litter,snag,log,soil)

NEP2005<-diff(rowSums(C2005),1)

sn.f<-0.116+rbeta(1000,2.6111,74)

sn.f<-ifelse(sn.f<0,0.116,sn.f)

#Simulation drawing from a distribution of snag fall rates that is consistent with the posterior distribution from the empirical model

#An extra zero is necessary relative to the simple computation because rbind demands a matrix as input

living<-append(living,0,after=0)

snag<-matrix(rep(0,2000),2,1000)

for (i in 3:302){

sn<-snag[i-1,]*(1-sn.f-sn.k)+sn.m*living[i]

snag<-rbind(snag,sn)

}

log<-matrix(rep(0,3000),3,1000)

for (i in 4:302){

lg<-log[i-1,]*(1-sf-lg.k)+sn.f*snag[i-1,]

log<-rbind(log,lg)

}

soil<-matrix(rep(0,3000),3,1000)

for (i in 4:302){

s<-soil[i-1]*(1-sk)+(log[i-1,]+litter[i-1])*sf

soil<-rbind(soil,s)

}

#making the sensitivity array

ca2050<-array(c(snag[-1,],log[-1,],soil[-1,]),dim=c(301,1000,3))

cb2050<-apply(ca2050,c(1,2),"sum")

C2050<-living[-1]+litter+cb2050

NEP2050<-diff(C2050,1)

deltaNEP<-(NEP2005-NEP2050)/NEP2005
