# Supplementary figures and images for: When a tree falls: Controls on wood decay predict standing dead tree fall and new risks in changing forests
Source: PLoS One. 2018 May 9;13(5):e0196712. doi: 10.1371/journal.pone.0196712 (PMC5942820; doi:10.1371/journal.pone.0196712)

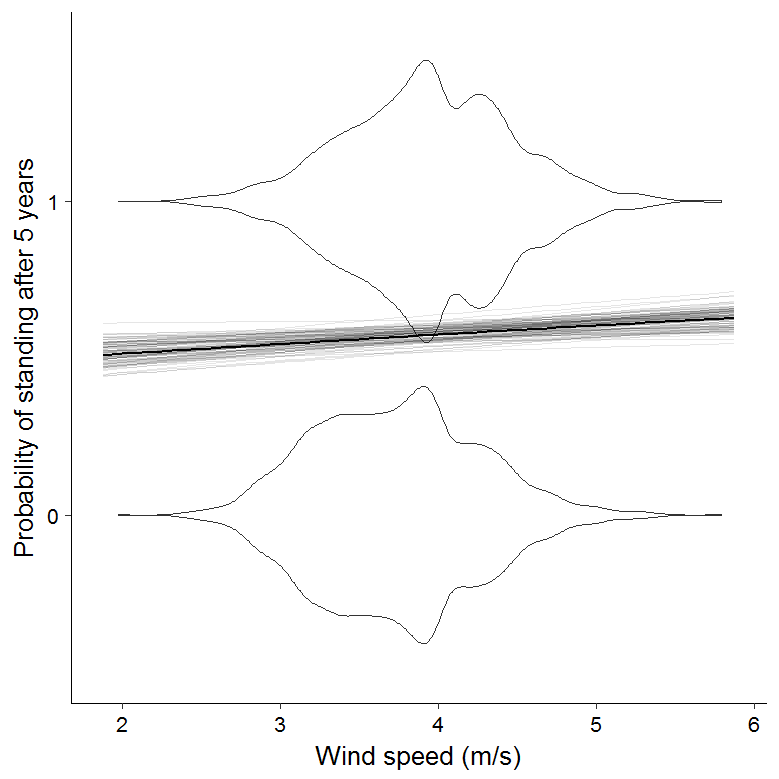

Supplement: S1 Fig — Thick central curve corresponds to the posterior mean for the effect of average wind speed at 10m on decay class 2 snag persistence and the transparent curve overlay represents uncertainty by showing 100 curves drawn from the posterior distribution of the relevant parameters. The vertical spread of the violin plots represents the distributions of the predictor values for standing (S = 1) versus fallen trees (S = 0). (TIFF) [file pone.0196712.s004.tiff]

Observed Proportion Standing by Species

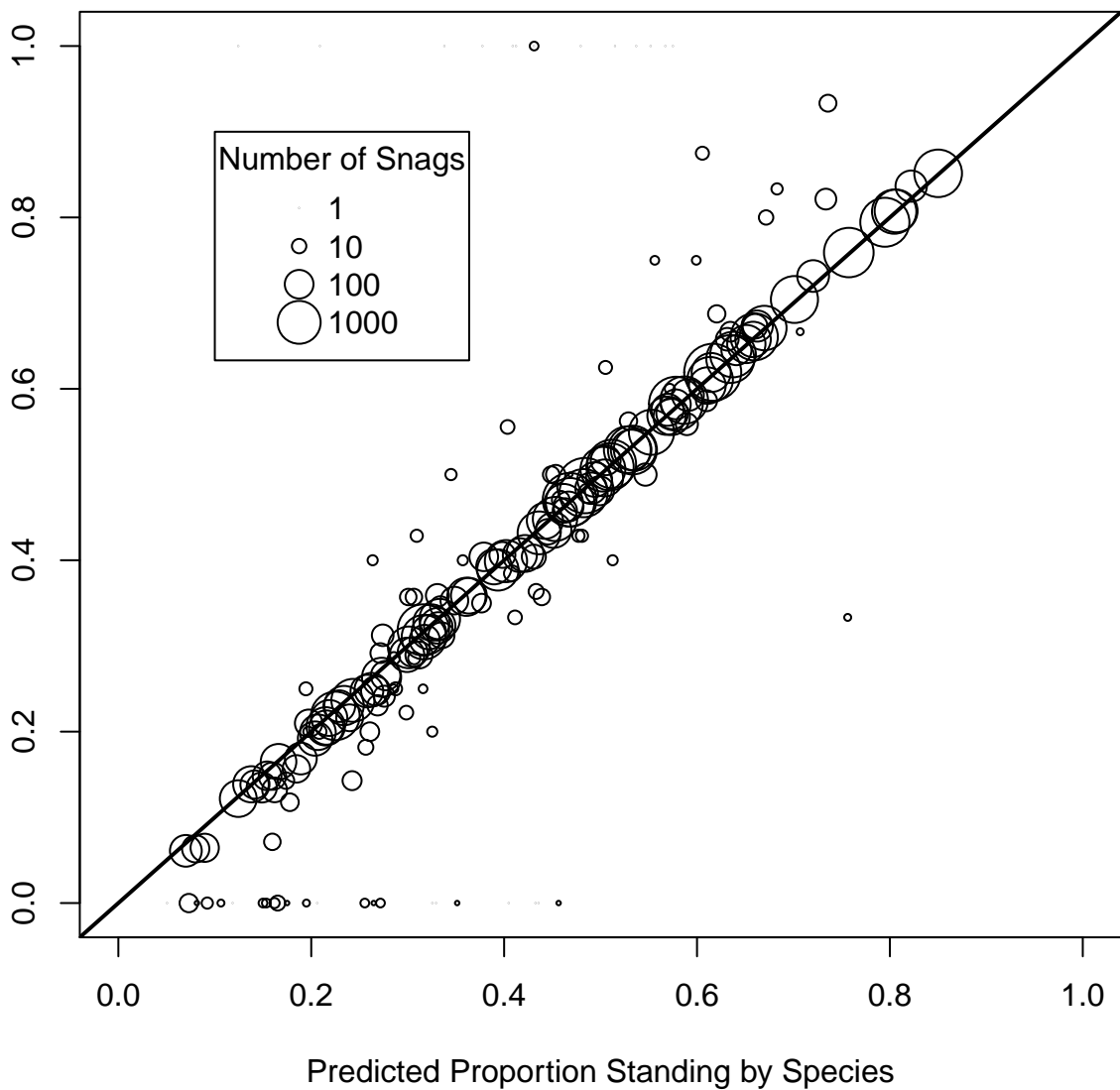

Supplement: S2 Fig — Symbol diameter is scaled by species abundance. (PDF) [file pone.0196712.s005.pdf]

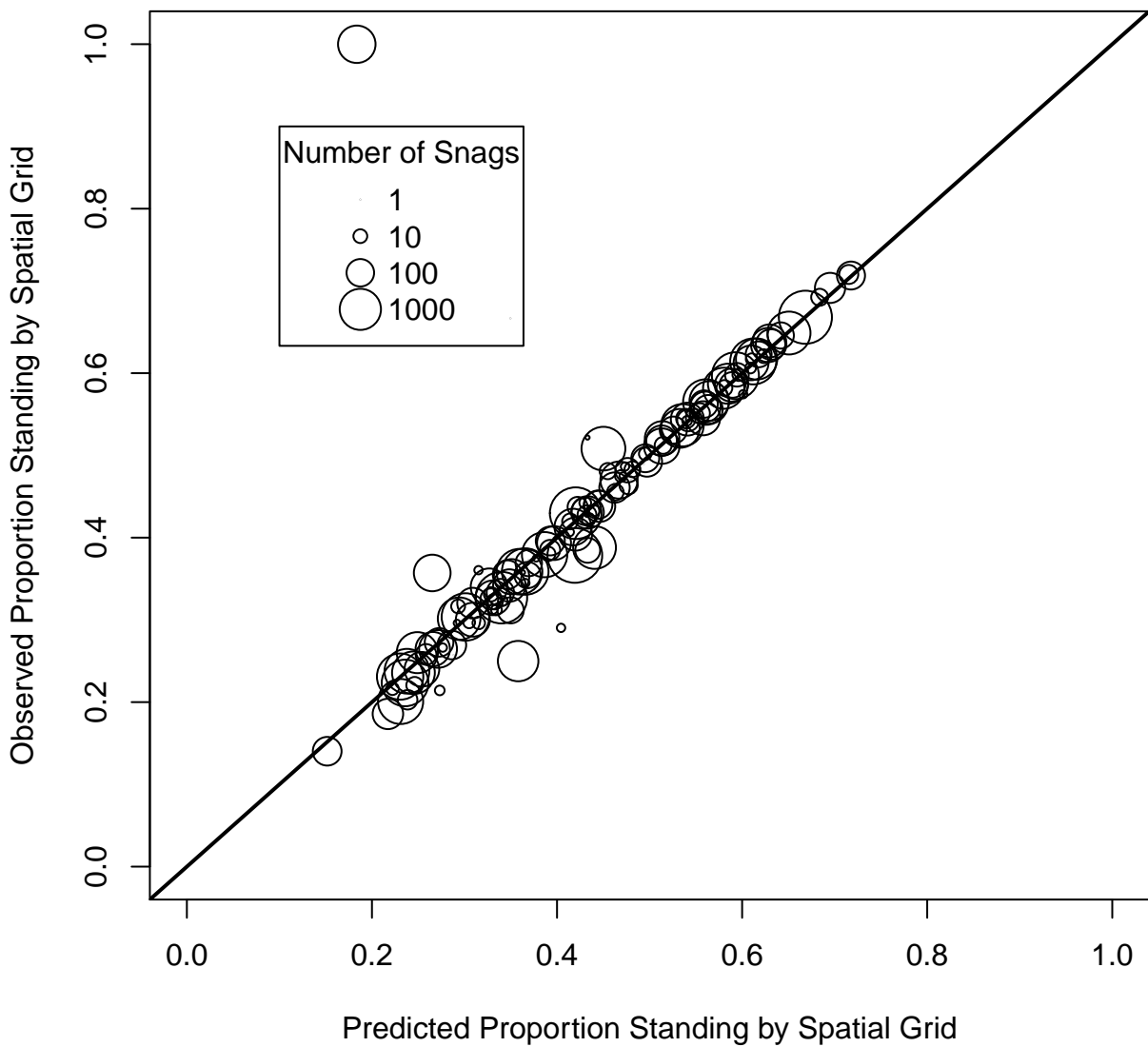

Supplement: S3 Fig — Symbol diameter is scaled by the abundance of snags per grid cell. (PDF) [file pone.0196712.s006.pdf]

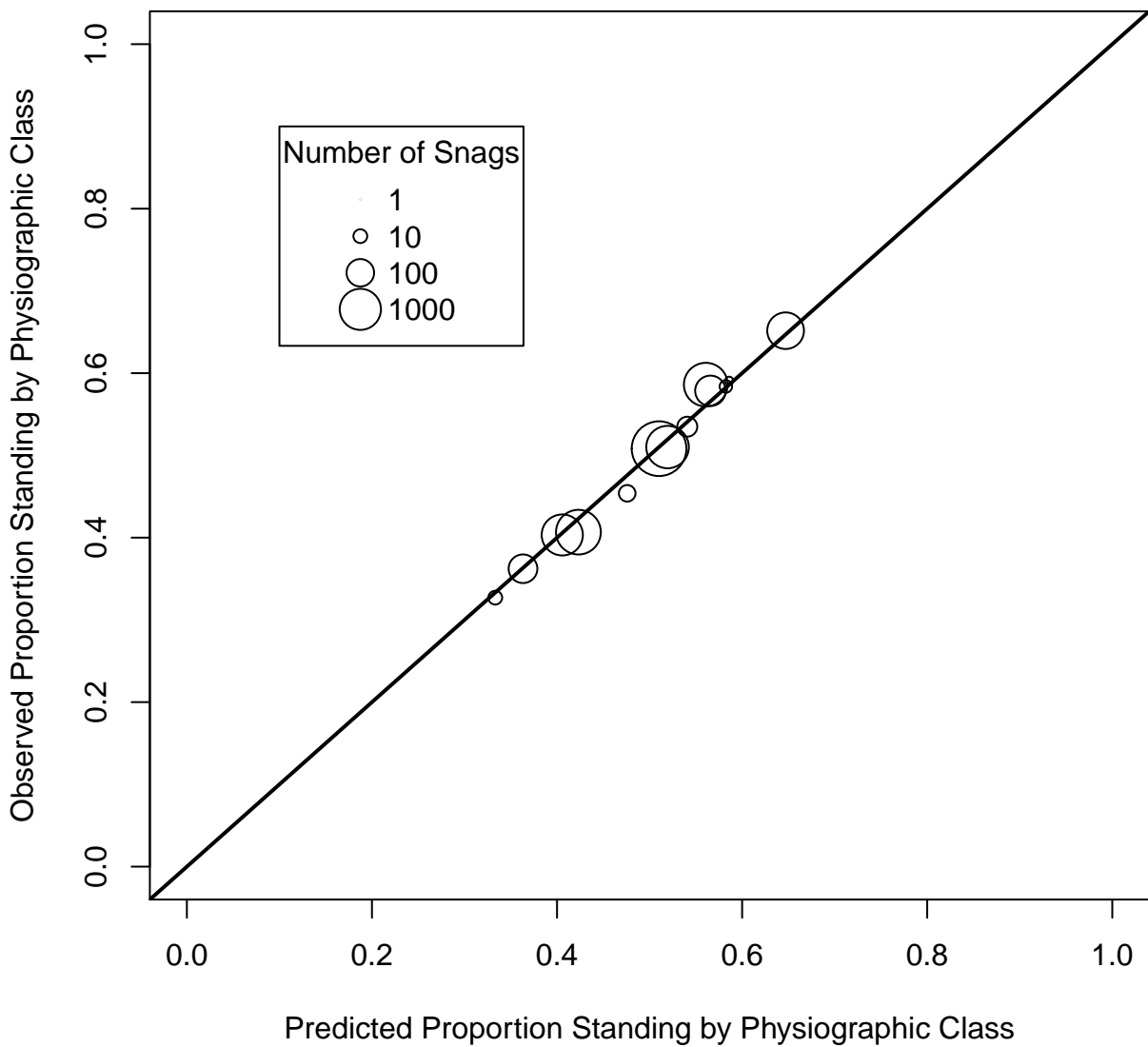

Supplement: S4 Fig — Symbol diameter is scaled by the abundance of snags per physiographic class. (PDF) [file pone.0196712.s007.pdf]
