# Supplementary material for: When a tree falls: Controls on wood decay predict standing dead tree fall and new risks in changing forests
Source: PLoS One. 2018 May 9;13(5):e0196712. doi: 10.1371/journal.pone.0196712 (PMC5942820; doi:10.1371/journal.pone.0196712)
Supplement: S1 Table — The final column indicates subsets of the data where the 95% CI for the effect excluded 0. (DOCX) [file pone.0196712.s008.docx]

**Table S1:** Snags occurring in physiographic settings with loose or disturbed soils are significantly more likely to fall compared to snags in settings with standing water. The final column indicates subsets of the data where the 95% CI for the effect excluded 0.
